# The Impact of Gut Microbiota-Derived Metabolites on the Tumor Immune Microenvironment

**DOI:** 10.3390/cancers15051588

**Published:** 2023-03-03

**Authors:** Maik Luu, Burkhard Schütz, Matthias Lauth, Alexander Visekruna

**Affiliations:** 1 Lehrstuhl für Zelluläre Immuntherapie, Medizinische Klinik und Poliklinik II, Universitätsklinikum Würzburg, 97080 Würzburg, Germany; 2 Institute of Anatomy and Cell Biology, Philipps-University Marburg, 35037 Marburg, Germany; 3 Department of Gastroenterology, Center for Tumor and Immune Biology (ZTI), Philipps-University Marburg, 35043 Marburg, Germany; 4 Institute for Medical Microbiology and Hygiene, Philipps-University Marburg, 35043 Marburg, Germany

**Keywords:** tumor microenvironment (TME), commensal bacteria, intratumoral microbiota, oncobiome, microbiota-derived metabolites, cancer immunotherapy

## Abstract

**Simple Summary:**

The tumor microenvironment (TME) comprises various non-malignant cells and soluble factors that surround cancer cells and which have mostly a pro-tumorigenic role. Growing evidence indicates that commensal bacteria are involved in the pathogenesis and progression but also in the suppression of various human cancers. Recently, bacterial communities that populate solid tumors have been described. This review provides insights into the complex interaction between gut-microbiota-derived metabolites and the cells of the TME. Novel studies indicate that some microbial molecules can be therapeutically exploited to enhance intratumoral immune responses and to improve the efficacy of cancer immunotherapies.

**Abstract:**

Prevention of the effectiveness of anti-tumor immune responses is one of the canonical cancer hallmarks. The competition for crucial nutrients within the tumor microenvironment (TME) between cancer cells and immune cells creates a complex interplay characterized by metabolic deprivation. Extensive efforts have recently been made to understand better the dynamic interactions between cancer cells and surrounding immune cells. Paradoxically, both cancer cells and activated T cells are metabolically dependent on glycolysis, even in the presence of oxygen, a metabolic process known as the Warburg effect. The intestinal microbial community delivers various types of small molecules that can potentially augment the functional capabilities of the host immune system. Currently, several studies are trying to explore the complex functional relationship between the metabolites secreted by the human microbiome and anti-tumor immunity. Recently, it has been shown that a diverse array of commensal bacteria synthetizes bioactive molecules that enhance the efficacy of cancer immunotherapy, including immune checkpoint inhibitor (ICI) treatment and adoptive cell therapy with chimeric antigen receptor (CAR) T cells. In this review, we highlight the importance of commensal bacteria, particularly of the gut microbiota-derived metabolites that are capable of shaping metabolic, transcriptional and epigenetic processes within the TME in a therapeutically meaningful way.

## 1. Introduction

Multiple lines of evidence suggest an essential role for the mutualistic interaction between intestinal microbiota and the host for the maturation of the immune system and maintenance of human health [1]. Long-lasting and parallel co-evolutionary processes have led to the establishment of a stable gut microbial ecology that exhibits reciprocal communication with the host [2]. The development of a protective immune system coincides with the expansion and alterations of the intestinal microbiota that, during the short weaning period, imprints the resistance or susceptibility to inflammatory processes later in life. This so-called “weaning reaction” is a central factor for the induction of Foxp3^+^ regulatory T cells (Tregs) in the gut and protection against diverse inflammatory and autoimmune diseases later in life [3]. Over the past decade, a number of studies have shown that the gut microbiota is not only essential for the mucosal tissue-associated development of the local immune system, but it also modulates the course of carcinogenesis and impacts treatment response [4,5], which may offer novel opportunities for the development of microbiota-based therapeutic strategies in the coming years. Emerging data demonstrate a complex interplay of bacterial and fungal molecules with cells of the tumor microenvironment (TME) across diverse cancer types [6,7]. There is evidence now that specific members of gut microbiota influence the treatment approaches, such as immune checkpoint inhibitors (ICI) and chimeric antigen receptor (CAR) T cell therapies [8,9,10,11]. The TME comprises various non-malignant cellular populations, such as tumor-infiltrating immune cells, fibroblasts and endothelial cells. Metabolic and transcriptomic alterations, induced by intercellular interactions, soluble factors and metabolites, frequently promote an immunosuppressive phenotype of immune cells, e.g., tumor-associated macrophages (TAMs), infiltrating myeloid-derived suppressor cells (MDSCs) and Tregs, which ultimately supports tumor progression and metastases [12]. Cancer and stroma cells commonly induce the expression of programmed cell death ligand 1 (PD-L1) that binds to programmed cell death 1 (PD-1) on T cells and leads to their exhaustion, a known phenomenon during cancer development and in chronic viral infections [13,14]. Recently, the antibodies targeting PD-L1, or its receptor PD-1, have revolutionized therapeutic options for the treatment of cancer patients [15,16]. Although ICI-based immunotherapy has greatly improved the overall survival among patients with metastatic melanoma, in other cancer types, only a small subset of patients responds to this treatment [17]. Remarkably, some commensal bacteria, such as *Akkermansia muciniphila* and *Bifidobacterium longum*, seem to augment anti-tumor immunity and enhance the effectiveness of ICI therapy [4,18,19,20,21]. Novel data suggest that the high diversity and richness of commensal bacteria synergize with ICI treatment and that exposure to antibiotics may result in worse outcomes among cancer patients [22,23]. Of note, the most commonly used laboratory mouse strain C57BL/6, reconstituted with natural microbiota of a wild population of mice (trapped in Maryland, USA), exhibited reduced tumor numbers in mutagen- and inflammation-induced colorectal tumorigenesis as compared to specific-pathogen-free (SPF) control mice [24,25], suggesting for yet uncharacterized, protective mechanisms due to natural host-microbiota crosstalk, which is absent in laboratory mice. By contrast, in some cancer types such as pancreatic cancer, host microbiota seems to have a pro-tumorigenic function by supporting the activity of immunosuppressive cells within the TME, such as TAMs and Tregs [26,27]. Thus, on one side, the commensal bacteria intimately linked to several human cancers are able to promote the course of carcinogenesis. On the other side, a beneficial microbial signature is associated with an increased response to ICI therapy and a better survival of patients. These findings highlight the importance of microbiota as a novel and still partially therapeutically unexploited factor, being potentially able to modulate cancer therapy and anti-cancer immunity. This review will focus on the emerging evidence of the functional impact of diverse microbiome-derived molecules on the cells of the tumor immune microenvironment.

## 2. The Intestinal Microbiota and Its Relation to Cancer Development and Cancer Immunotherapy

Progress in both basic cancer research in experimental animal models and translational oncology has essentially contributed to the current understanding of how gut commensal bacteria impact cancer development and targeted therapy for cancer. Mutual interactions between intestinal microbiota and host T cells seem to be a key factor that contributes substantially to a bacteria-primed immune reaction and the trafficking of intestinal and circulating T cells to tumor tissue that supports cancer therapy [28]. There is a growing awareness of the role of a “favorable” microbiota composition that correlates with an efficient response to ICI treatment in humans and mice [29]. Using a murine model of ICI therapy (anti-cytotoxic T-lymphocyte-associated protein-4 (CTLA-4) blockade), Vetizou et al. found that enhanced anti-cancer immunotherapy relies on the presence of *Bacteroides fragilis* or *Bacteroides thetaiotaomicron* within the gut microbiome [30]. Another study suggested a strong impact of the *Bifidobacterium* species on the infiltration of intratumoral CD8^+^ T cells, which resulted in enhanced efficacy of anti-PD-L1 immunotherapy. A subsequent report demonstrating the abundance of eight different commensal species with a better response to ICI therapy confirmed the association of *Bifidobacterium longum* and an augmented anti-PD-1 efficacy [31]. Importantly, the fecal microbiota transplantation (FMT) from human responders to ICI therapy led to reduced tumor growth, an increasing number of intratumoral CD8^+^ T cells and the enhanced efficacy of PD-1/PD-L1 blockade in mice [32,33,34]. Similarly, a recent study has revealed that a defined commensal consortium comprising 11 human bacteria that were derived from the feces of healthy human donors elicits CD8^+^ T cell responses and promotes anti-tumor effects in murine subcutaneous tumor models [35,36]. Interestingly, also “non-favorable” members of gut microbiota, such as *Roseburia intestinalis* and *Ruminococcus obeum*, have been recently identified [31]. Collectively, the composition of gut microbiota influences anti-cancer immune responses, tumor microenvironments and the clinical benefits of ICI therapy. Although commensal bacteria are capable of reshaping the functionality of cells surrounding the tumors and even of enhancing the efficacy of anti-tumor immunity, our understanding of the impact of specific microbiota-derived species and their molecules on the tumor immune microenvironment is still limited. Several mechanisms have been suggested, potentially explaining how gut bacteria may influence anti-cancer immune surveillance and TMEs. The system effects of gut microbes can be mediated via the ligands of pattern recognition receptors that deliver adjuvant signals for the cells of innate immunity, such as dendritic cells and macrophages [37]. Additionally, cross-reactive anti-tumor T cell responses can be generated by specific T cells that recognize microbial antigens with high similarity in their structure to tumor neoantigens [38,39]. Finally, the host/microbiota interactions can be mediated through small molecules produced by commensal bacteria that can leave the bacterial community in the intestine and reach the TME via circulation [40,41,42,43]. Recent studies have demonstrated that gut microbiota-derived metabolites are capable of eliciting and strengthening T cell-mediated anti-tumor immunity [44,45].

## 3. The Oncobiome and Cancer

Reduced diversity or altered composition of the intestinal microbiome has been found to correlate with many chronic disorders, such as metabolic dysfunctions and cardiovascular, inflammatory and autoimmune diseases [46]. Generally, a more diverse gut microbiome has a positive effect on the functional diversity of the immune system, likely lowering the risk of developing cancer. For example, the diversity of the microbial community is an independent predictor of survival in cervical cancer [47]. It was observed that cancer patients with a high diversity of gut microbiota had increased tumor infiltration of Th1 and CTLs in various cancer types. Surprisingly, a novel study investigating the human tumor microbiome uncovered that intratumoral bacteria are present in various solid tumors, such as breast and ovarian cancer, lung and pancreatic tumor tissues, and even in tumors that have no direct communication with the external environment (e.g., glioblastoma or bone tumors) [6,48]. Diverse intracellular bacteria have been detected mostly in both cancer and the neighboring immune cells. The characterization of the tumor microbiome revealed that different tumor types have distinct bacterial compositions. Interestingly, at the phylum level, only two phyla (Firmicutes and Proteobacteria) have been mostly observed in the TME; however, the Proteobacteria to Firmicutes ratio seems to vary between cancer types. Furthermore, a high diversity was found for bacterial families, genera and species among various cancers [6]. Several mechanisms may be involved in the translocation and transport of bacteria to the TME during tumor development. A leaky and flexible vasculature may allow the entry of circulating bacteria and immune cells, such as macrophages, engulfing and transporting bacteria to tumor tissue. Currently, it is difficult to speculate whether intratumoral bacteria actively modulate the development of cancer or if bacteria appear at later stages in established tumors, where they can persist in certain niches. A very recent study suggests that the distribution of bacteria in the TME does not occur randomly. Instead, the presence of tumor-associated bacteria in immunosuppressive microniches points to a highly organized colonization of tumor tissues that affect the behavior of tumor and immune cells [49]. Intriguingly, it was postulated that the cell-associated members of the intratumoral microbiota could drive the migration of cancer cells and impact the cellular heterogeneity of the TME. Interestingly, the total bacterial load in tumors was negatively regulated with the expression of tumor suppression protein p53 [49]. Our better understanding of these effects may contribute to the development of alternative approaches to enhance the current cancer treatment efficacy by modulating the composition of the so-called oncobiome [50]. The presence of tumor-associated bacteria in colorectal carcinoma is probably easier to explain than in cancers that are not in close proximity to the intestinal microbiome. The processes that damage the integrity and function of the epithelial barriers in our body might compromise mucosal homeostasis, leading to microbial dysbiosis. Interestingly, intestinal bacteria and some oral bacteria have been found in colorectal cancer (CRC) samples. It was reported that *Fusobacterium nucleatum*, a common oral bacterium, can migrate to the colon, where it enriches in tumor tissue and impairs the therapeutic outcome and prognosis of radiotherapy and promotes colorectal carcinogenesis [51,52,53,54]. Transcriptional modification, induced by this invasive bacterium, has been related to the upregulation of signaling cascades triggered through the growth factor receptors, such as epidermal growth factor receptor (EGFR) and platelet-derived growth factor (PDGF), as well as NF-kB signaling, while pathways linked to the cell cycle, DNA damage repair and p53 signaling were downregulated. In cultured cancer cell spheroids treated with *F. nucleatum*, intestinal epithelial cells detached from the spheroid mass and infiltrated the surrounding collagen [49]. Notably, this member of the oral microbiota was also abundantly detected in breast and pancreatic tumor patient cohorts [55,56]. Furthermore, using advanced high-throughput 16S rRNA sequencing techniques, several studies have demonstrated that pancreatic tumor cohorts are enriched in Proteobacteria, which are normally found in duodenum tissues [57,58]. These findings suggest a retrograde bacterial translocation from the duodenum to the pancreatic duct. Of note, in both cancer types with high frequencies of *K*-*Ras* mutations, *pancreatic* adenocarcinoma (PDAC) and lung adenocarcinoma, the intratumoral microbiota promotes the development of cancer due to local microbiota-immune crosstalk and by modulating the tumor immune microenvironment [59,60,61]. Interestingly, not only bacteria but also pancreatic fungal mycobiome seem to promote oncogenesis. Mechanistically, the binding of glycans of the fungal wall to the mannose-binding lectin (MBL) accelerates oncogenic progression [62]. Following diagnosis, the actual five-year survival of PDAC patients is very low (approximately 9%). A recent study focusing on the tumor-associated microbiota in short-term survivors and long-term survivors offered new insights into a complex interaction between bacterial communities and the cells of the TME in PDAC. In the tumor tissue of long-term survivors, particularly three genera (*Saccharopolyspora*, *Pseudoxanthomonas* and *Streptomyces*) were enriched that were marginally present in short-term survivors. A strong correlation between these top-three genera and CD8^+^ and granzyme B^+^ densities was found for long-term survivors [63], suggesting that infiltration of the TME with CTLs, but also higher activity of these cells might be connected to a specific microbial signature within tumor tissue. Collectively, although it is premature to interpret the functional influence of the local microbiome composition within tumors, the targeted modulation of tumor-associated bacteria may affect the effectiveness of cancer treatment. It might be important to define a specific fraction of bacteria that belong to a “favorable oncobiome” with the potential to reshape tumor immune responses and “re-educate” the cells of the TME. In the future, such therapeutic approaches could be combined with established types of cancer immunotherapies, such as CAR-T cell or ICI therapy. The discovery of specific tumor-associated microbiome signatures in various human cancer types may also lead to the development of novel diagnostic tools to predict the effectiveness of cancer immunotherapies.

## 4. Gut-Microbiota-Derived Molecules and Cancer

An important functional aspect of the host-microbiome crosstalk is determined by a variety of bacterial enzymatic systems that are capable of synthetizing a plethora of small molecules, potentially being able to exert direct effects not only in the intestine but also to modulate the function of cells in remote organs [64]. In contrast to commensal bacteria that are predominantly located in the luminal compartment of the large intestine and caecum, small molecules derived from the microbiome can easily cross the epithelial layer and diffuse through the lamina propria to enter the systemic circulation. Several studies have detected plenty of microbial molecules in the human bloodstream, estimating that between 5 and 10% of all plasma metabolites are derived from gut microbiota [65]. For a long time, the products generated by gut bacteria were considered merely dead-end by-products of their metabolic pathways [66]. However, in the past decade, small molecules produced by commensals have received increased attention in cancer research. Novel findings have challenged the long-held “metabolic waste dogma”, indicating a crucial role for microbiota-derived metabolites in communication with host cells [41,67], thus also potentially being able to influence the TME. The microbial signals mediated via the secretion of small metabolites and bacterial membrane-associated factors are thought to play a central role in the functional shaping of the immune system [68]. With a better understanding of complex intestinal microbial communities in our gut in the last decade, it becomes clear that various molecular families synthetized by luminal microbes are involved in the communication with host T cells. Commensal bacteria-derived metabolites, such as short-chain fatty acids (SCFAs) and secondary bile acids, are unique bioactive compounds that play an important role in the regulation of the differentiation of T cells into various specialized subsets, including the Th17 cells and Tregs that are essential for intestinal immune homeostasis [69,70,71]. Th17-derived cytokines, IL-22 and IL-17A, reinforce barrier function at the steady state by promoting epithelial regeneration and the expression of antimicrobial peptides, while cytokine IL-10, secreted from Tregs and other immune cells, prevents intestinal inflammation [72]. Immune imbalances caused by disrupting the epithelial barrier and intestinal homeostasis lead to pathological outcomes, such as inflammatory bowel disease (IBD) and colitis-associated colorectal cancer [73].

**Figure 1:**
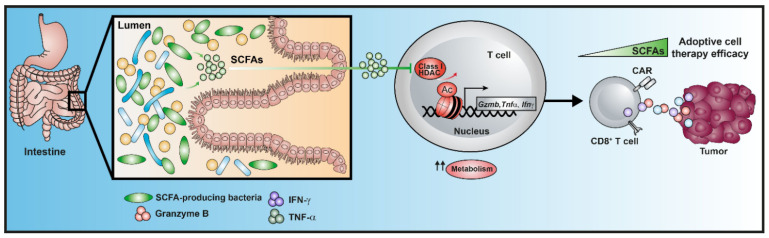
Schematic overview of the molecular mechanisms underlying modulation of T cell function and phenotype by SCFAs. The SCFAs butyrate and valerate seem to be good candidates for augmenting the efficacy of cellular immunotherapy for cancer. These microbial metabolites enhance the effector cytokine production and functionality of CD8^+^ T cells by fine-tuning their metabolic and epigenetic signatures. Abbreviations: short-chain fatty acids (SCFAs); chimeric antigen receptor (CAR); granzyme B (Gzmb); tumor necrosis factor (TNF); interferon (IFN).

Although there is increasing evidence to suggest an influence of bacterial metabolites on tumor development, mechanisms underlying a direct interaction between the microbial molecules and cells of the tumor microenvironment are still poorly understood. Intestinal commensal bacteria have an enormous genetic and chemical diversity, outnumbering their host genome by more than 25-fold regarding genetic composition [74]. Anaerobic fermentation of dietary fiber in the gut lumen by commensal bacteria leads to the generation of SCFAs, the most abundant class of microbial metabolites comprising carboxylic acids with aliphatic tails of 1–5 carbons [75]. Although microbial fermentation of dietary indigestible carbohydrates is the largest source of SCFAs, some branched SCFAs (BCFAs), such as isobutyrate and isovalerate, can be generated from amino acids by bacterial utilization of valine and leucine [75]. Bacterial SCFAs, such as acetate (C2), propionate (C3), butyrate (C4) and valerate (C5), are potent signaling molecules that promote the induction of mucosal protective IgA responses and the epithelial barrier function [76,77]. Moreover, SCFAs are the first important example of how microbiota-derived molecules can regulate anti-cancer immunity and cancer immune surveillance [78]. Recently, we demonstrated that butyrate and valerate enhanced the cytotoxic capacity of murine and human CTLs by increasing the activity of the mTOR complex and by inducing the expression of granzyme B, which is the key death-inducing effector molecule for a potent anti-cancer immunity. Notably, both SCFAs and the valerate-producing bacterium *Megasphaera massiliensis* (a low-abundant commensal isolated from human gut) substantially increased CTL-mediated anti-tumor immunity in vivo, which resulted in reduced tumor growth in experimental models of melanoma and pancreatic cancer [45]. By acting as a potent physiological histone deacetylase (HDAC) inhibitor of class I HDACs and by enhancing the metabolism and functional activity of CTLs, SCFAs might be a potential therapeutic candidate to improve the adoptive T cell transfer in various tumors. Novel data from our laboratory suggest that the treatment of human CAR-T cells with SCFAs enhances their efficacy and ability to kill cancer cells in an in vitro killing assay by increasing their secretion of effector cytokines TNFα and IFN-γ (Figure 1).

While the effects of SCFAs on anti-tumor immunity, either by directly impacting the T cells or indirectly influencing antigen-presenting cells, are well documented [79], much less is known about the potential influence of other microbiome-derived metabolites. Apart from SCFAs, various bacterial molecules, such as secondary bile acids, various oligosaccharides, peptidoglycan fragments, tryptophan catabolites, inosine and polyamines are capable of modulating the cells of the immune system [40]. Polysaccharide A (PSA) of *Bacteroides fragilis* was previously shown to interact directly with dendritic cells and to promote immune regulation of the T cells via TLR2 [80,81]. Recently, one study investigated the impact of microbiota-derived inosine on the outcome of ICI therapy. In this study, inosine strongly enhanced the efficacy of ICI therapy in several experimental tumor models by modulating T cells via adenosine A2A receptors [82]. The oral administration of inosine or treatment of mice with the inosine-producing bacterium *Bifidobacterium pseudolongum*, together with anti-CTLA blockade, resulted in a significantly reduced tumor mass and an increase in the frequency of IFN-γ-producing Th1 cells. Remarkably, some dietary compounds, particularly polyphenols, have been suggested to modulate the composition of intestinal microbiota, which has a significant influence on anti-tumor immunity. It was shown that castalagin, an ellagitannin derived from the polyphenol-rich berry camu-camu (*Myrciaria dubia*), supported the anti-PD-1 activity by expanding the commensal bacteria associated with strong immunotherapy responses, such as *Ruminococcaceae* and *Alistipes* [83].

Of note, not only beneficial effects of gut microbiota-derived molecules on the tumor microenvironment have been described. A very recent paper by Hezaveh et al., investigated the influence of dietary tryptophan on the development of PDAC [84]. This essential amino acid, tryptophan, serves as a substrate for several enzymes within the gut microbiota community. Various commensals can convert dietary tryptophan into multiple derivatives that may impact T cells and macrophages via the aryl hydrocarbon receptor (AhR) [41]. Interestingly, the AhR activity in the TAMs of the PDAC microenvironment was dependent on the metabolization of dietary tryptophan to indoles by Lactobacillus species in the gut lumen [84]. Removing tryptophan from the diet resulted in reduced TAM-associated AhR activity and increased the infiltration of TNFα^+^IFNγ^+^CD8^+^ T cells into the TME. In addition, increasing evidence suggests that secondary bile acids, which are produced solely by intestinal bacteria, can induce DNA damage and modulate the tumor’s immune microenvironment in CRC [43]. Moreover, colibactin-producing *Escherichia coli* strains, which are frequently found to colonize CRC lesions, can also induce DNA damage in epithelial cells [85]. Finally, novel preclinical reports indicate dual effects for SCFAs in tumor biology. By investigating the gut metabolite changes associated with the progression of CRC, the SCFA formate and the BCFA isovalerate were identified as oncometabolites that contribute to the invasion of cancer cells and metastasis [86,87]. A comprehensive overview of the known interactions between small microbial molecules and the TME, which either might act as a target of cancer therapy, or can promote tumor growth, is summarized in Table 1. Collectively, although the novel results suggest that microbial metabolites have a potential to directly influence complex and dynamic processes that dictate the progression and invasion of tumors, a substantial amount of exploratory work will be required in the future to better understand how microbiota-derived molecules promote their effects on the cells in the tumor immune microenvironment.

## 5. Challenges Associated with Therapeutic Potential of Microbial Metabolites

Tumors develop gradually in a complex interaction with various cellular components surrounding the tumor mass, such as stroma cells, endothelial cells, adipocytes and immune cells, most of which exhibit an immunosuppressive capacity and collaborate with cancer cells to evade immune surveillance. Currently, several therapeutic strategies have been employed to disrupt the crosstalk of tumors with cancer-associated fibroblasts and other cells in the TME. Recently, the enhancing effects of the SCFAs butyrate and valerate on anti-tumor immunity and the microenvironmental architecture of solid tumors have been described [45]. These findings pave the way for the translational progression of laboratory studies to novel therapeutic interventions. It is tempting to speculate that many other microbiota-derived components and metabolites might be able to influence the anti-cancer activity of immune cells by modulating the TME. However, several questions still remain open, and particularly more refined delivery strategies to exploit the therapeutic potential of gut microbiota-derived molecules are needed. Oral administration of SCFAs is only moderately efficacious and is associated with an unpleasant odor and rapid absorption and oxidation. In order to address this problem, several novel approaches have been developed. Oral supplementation of butyrate in a starch-conjugated form was shown to have beneficial effects in suppressing type 1 diabetes [93]. In this study, high amylose maize starch (HAMS), which resists digestion in the upper gastrointestinal tract (GI), was used. Chemical modification, such as propionylated and butyrylated HAMS, allowed an effective delivery of esterified propionate or butyrate to the colon and other organs of mice. In a second approach, to overcome the existing limitations, water-soluble micelles carrying butyrate in their core were applied to deliver high amounts of butyrate to the lower GI to protect mice from colonic inflammation [94]. Such novel techniques could soon be tested in experimental tumor models to try to achieve therapeutic effects and the efficient biodistribution of SCFAs in the body.

Interestingly, some bioactive molecules produced by commensals may have opposing roles in regulating important physiological aspects of the host. The small bacterial molecule trimethylamine (TMA), which is produced by the gut microbiome, is rapidly absorbed into the circulatory system and thereafter oxidized to trimethylamine N-oxide (TMAO) in the liver. Increased blood levels of TMAO were found to be associated with an increased risk for atherosclerosis [95]. A novel study suggested a potential role for this molecule in the tumor immune microenvironment of triple-negative breast cancer (TNBC). TMAO was abundant in tumors with an activated immune microenvironment and promoted anti-tumor immunity in TNBC [90], providing a further example of microbial metabolite-immune crosstalk, which can be exploited for therapeutic strategies to enhance the efficacy of cancer immunotherapy. Targeted delivery of such drug-like molecules to tumors may be achieved by using nanoparticle-based technologies while minimizing possible adverse systemic effects. Paradoxically, lactate, a key metabolite produced by glycolysis and highly abundant in the TME, in which it induces the M2-like polarization of TAMs and supports tumor growth [96], appears to increase the stemness of CD8^+^ T cells and augment anti-tumor immunity [97]. Several commensal bacteria are able to generate D-lactate, which is the stereoisomer of L-lactate and not produced by eukaryotic cells. In the future, it would be important to test the role of both lactate isomers in influencing cellular therapy and the anti-cancer capacity of CAR-T cells. Such small molecules might be ideal drug candidates for the in vitro treatment of CAR-T cells or CTLs before introducing them into patients by intravenous infusion to potentially enhance their ability to attack cancer cells. Taken together, while characterizing novel molecules derived from human gut microbiota is a promising area for discovering novel drug candidates, more fundamental laboratory research will be needed to expand the current cancer treatment options.

## 6. Conclusions

A large heterogeneity within the TME, with regard to the composition of various cell types surrounding the tumor cells and their spatial distribution, is one of the major obstacles compromising cancer treatment outcomes. Different immune cell types are involved in pathological and immunosuppressive processes in the TME. In addition, a continually emerging body of evidence supports the role of various commensal bacteria and their metabolic products in either promoting tumor development or augmenting cancer immunotherapy. Novel studies suggest that microbial SCFAs are capable of modulating the cellular architecture of the TME by triggering anti-tumor T cell responses and that bacterial molecules, such as inosine or TMAO, enhance the efficacy of targeted immunotherapies, such as ICI therapy. In contrast, some microbial tryptophan derivatives synthetized by intestinal bacteria rather support the pro-tumorigenic function of TAMs in PDAC. Further research is required to characterize novel, still unknown microbiota-derived molecules that may be able to act on the cells of the tumor immune microenvironment, which could be a central translational step for the development of novel microbiota-based interventional strategies.

Although many challenges exist, which must be addressed to achieve these goals, an innovative strategy could focus on the design of patient-tailored cancer therapeutics by exploiting diverse microbiota-derived molecules. Various interdisciplinary approaches, ranging from microbiology, high-throughput sequencing techniques and comprehensive functional analysis of the whole gut bacterial genome to biotechnology, offer new insights into the transcriptional, metabolic and epigenetic networks within the human microbiome. Currently, many studies are attempting to translate these novel findings to the clinic to achieve optimal and targeted manipulation of the immunosuppressive cellular networks within the TME by small microbial molecules, which is probably one of the most promising therapeutic strategies to extend the current options for tumor therapy.

## Figures and Tables

**Table 1:** Overview of current cancer-specific studies that suggest either promotion of tumor progression or suppression of tumor growth by microbial metabolites. Abbreviations: pancreatic ductal adenocarcinoma (PDAC); colorectal cancer (CRC); azoxymethane (AOM); dextran sodium sulfate (DSS). Red color indicates tumor-promoting role, while blue color is used for tumor-suppressing capacity of metabolites.

Microbial Molecules	Cancer Type	References
***Formate***	CRC	Ternes D. et al., Nature Metabolism, 2022 [87]
***Acetate***	Melanoma	Qiu J. et al., Cell Reports, 2019 [88]
***Propionate***	Metastatic melanoma	Coutzac C. et al., Nature Communications, 2020 [89]
***Butyrate***	Adoptive transfer of MC-38 colon adenocarcinoma cells, melanoma, PDAC	He Y. et al., Cell Metabolism, 2021 [79]; Luu M. et al., Nature Communications, 2021 [45]
***Valerate***	Melanoma, PDAC	Luu M. et al., Nature Communications, 2021 [45]
***Isovalerate***	CRC	Yachida S. et al., Nature Medicine, 2019 [86]
***Insosin***	CRC (AOM /DSS model), adoptive transfer of colon adenocarcinoma cells	Mager LF et al., Science, 2020 [82]
***Trimethylamine-N-oxide***	Triple-negative breast cancer	Wang H. et al., Cell Metabolism, 2022 [90]
***Colibactin***	CRC	Iftekhar A. et al., Nature Communications, 2021 [91]
***Secondary bile acids***	CRC	Rial NS et al., International Journal of Cancer, 2009 [92]
***Tryptophan-derived indoles***	PDAC	Hezaveh K. et al., Immunity, 2022 [84]
